# Evolving Concepts in the Pathogenesis of NASH: Beyond Steatosis and Inflammation

**DOI:** 10.3390/ijms15058591

**Published:** 2014-05-14

**Authors:** William Peverill, Lawrie W. Powell, Richard Skoien

**Affiliations:** 1 Department of Gastroenterology and Hepatology, Royal Brisbane and Women’s Hospital, Brisbane 4029, Australia; E-Mails: william.peverill@health.qld.gov.au (W.P.); lawrie.powell@qimrberghofer.edu.au (L.W.P.); 2 The University of Queensland Centre for Clinical Research, Brisbane 4029, Australia; 3 QIMR Berghofer Medical Research Institute, Brisbane 4006, Australia; 4 School of Medicine, the University of Queensland, Brisbane 4006, Australia

**Keywords:** non-alcoholic fatty liver disease, non-alcoholic steatohepatitis, pathogenesis, fibrosis, lipotoxicity, inflammation, immune response, treatment

## Abstract

Non-alcoholic steatohepatitis (NASH) is characterised by hepatic steatosis and inflammation and, in some patients, progressive fibrosis leading to cirrhosis. An understanding of the pathogenesis of NASH is still evolving but current evidence suggests multiple metabolic factors critically disrupt homeostasis and induce an inflammatory cascade and ensuing fibrosis. The mechanisms underlying these changes and the complex inter-cellular interactions that mediate fibrogenesis are yet to be fully elucidated. Lipotoxicity, in the setting of excess free fatty acids, obesity, and insulin resistance, appears to be the central driver of cellular injury via oxidative stress. Hepatocyte apoptosis and/or senescence contribute to activation of the inflammasome via a variety of intra- and inter-cellular signalling mechanisms leading to fibrosis. Current evidence suggests that periportal components, including the ductular reaction and expansion of the hepatic progenitor cell compartment, may be involved and that the Th17 response may mediate disease progression. This review aims to provide an overview of the pathogenesis of NASH and summarises the evidence pertaining to key mechanisms implicated in the transition from steatosis and inflammation to fibrosis. Currently there are limited treatments for NASH although an increasing understanding of its pathogenesis will likely improve the development and use of interventions in the future.

## 1. Introduction

Non-alcoholic fatty liver disease (NAFLD) has become the most common chronic liver disease in Western populations [1] and its more severe form, non-alcoholic steatohepatitis (NASH), can result in progressive fibrosis and cirrhosis. The pathogenesis of NASH was originally conceptualised as a disease of consecutive hits: the accumulation of fat in the liver cells (steatosis) that sensitised the liver to a second metabolic insult triggering a cascade of tissue damage (inflammation) resulting in fibrosis. It is now appreciated that a more complex process involving multiple parallel metabolic hits is probably responsible for tissue injury and that other factors and pathogenic mechanisms are also likely to promote disease progression. Central to an understanding of the pathogenesis of NASH is the concept of lipotoxicity and the contributions of insulin resistance and oxidative stress to hepatocyte damage. Hepatocyte death and an ensuing inflammatory cascade likely represent the nexus between inflammation and fibrosis but cellular senescence may also play a role in disease progression. Recognition of the importance of the interactions between cells of the inflammatory response, probably representing a Th17 response, and other cell types within the liver (hepatocytes, hepatic stellate cells, and ductular components) has also contributed to our understanding of pathogenesis. This review seeks to provide an overview of these evolving concepts and assess their relative contributions and their potential application to the development of therapeutic agents.

## 2. The Evolution of Hypotheses of NASH Pathogenesis

Originally, the progression of NAFLD from simple steatosis to NASH was conceptualised as a “two hit” model [2]. It was suggested that hepatic steatosis as the first hit sensitises the liver parenchyma to a second hit in the form of a further metabolic stressor that initiated a cascade of inflammation, steatonecrosis, and fibrosis. Experts in the field since then have come to regard this model as an over-simplification of pathogenesis in NASH and believe that multiple parallel hits are more likely responsible for disease progression [3]. A myriad of potential insults have been proposed, including (but not limited to) impaired mitochondrial adenosine triphosphate (ATP) activity [4], depletion of mitochondrial glutathione [5,6], hypoxia associated with impaired blood flow or obesity-related obstructive sleep apnoea [7] dysregulated adipokine production [8], the effects of a high fructose diet [9] and rapid weight loss [10]. This disparate collection of insults affecting multiple areas of normal metabolism reflects the complex nature of this disease making identification of a “key” pathogenic mechanism unlikely. More recently, the contribution of histological changes associated with progressive injury, such as an (as yet) uncharacterised portal inflammatory infiltrate and the proliferation of hepatic progenitor cells (HPCs) and an accompanying ductular reaction (DR), have been studied. It now seems clear that progressive NASH is more sophisticated than simply the consecutive accumulation of hepatic fat causing inflammation and fibrosis, and the relative contributions of other pathogenic factors should be represented in newer models of NASH pathogenesis.

## 3. A Central Role for Lipotoxicity and Insulin Resistance

Current evidence suggests that “lipotoxicity” represents the major mechanism underlying hepatocyte dysfunction leading to disease progression in NASH. Lipotoxic injury appears to occur in the setting of excess free fatty acid (FFA) traffic, especially saturated fatty acids (SFAs), rather than due to simple triglyceride accumulation. This view is supported by studies that demonstrate that increased hepatocyte triglyceride formation [11] or reduced export of lipid [12] does not, of itself, increase inflammation. Furthermore, evidence suggests that triglyceride accumulation may actually be a protective mechanism to counter lipotoxicity [13,14]. A number of other studies have shown lipotoxic injury is reduced if alternative pathways of fatty acid (FA) disposal are available [15–18]. This suggests that triglycerides themselves are unlikely to be the cause of hepatocyte injury in NASH and probably occurs *in parallel* with the generation of toxic metabolites, with these lipotoxic metabolites being primarily responsible for disease progression [19].

**Figure 1.**
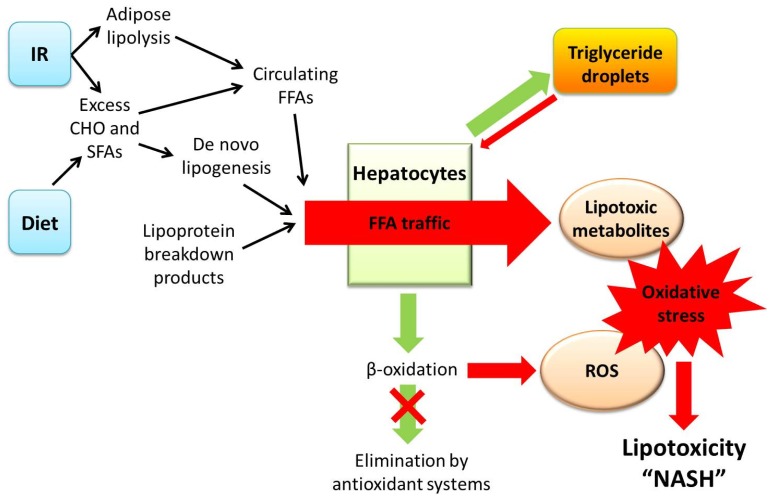
The lipotoxicity model of pathogenesis in non-alcoholic steatohepatitis (NASH). In the setting of established insulin resistance (IR) and a diet high in saturated fats, hepatic traffic of excess free fatty acids (FFA) induces hepatocyte injury via lipotoxicity, caused by oxidative stress through the generation of lipotoxic metabolites (such as ceramides, diacylglycerols, and lysophosphatidyl choline) and reactive oxygen species (ROS). Protective mechanisms are shown as green arrows and injurious mechanisms are shown as red arrows. In the current model, the accumulation of triglyceride within hepatocytes likely represents a protective adaptation to excess FFA traffic, with only a minor contribution to the toxic effects of FFA flux via autophagy. (In the previous model of pathogenesis, the accumulation of hepatocyte triglyceride (“steatosis”) was considered a pre-requisite for oxidative stress, whereas emerging data suggest that steatosis occurs in parallel with lipotoxicity). A diet high in carbohydrates (CHO) and saturated fatty acids (SFAs) contributes to the production of excess FFA, as does the development of insulin resistance. In the chronic disease state, safe disposal of FFA via beta-oxidation and cellular antioxidant systems is overwhelmed resulting in the accumulation of excess ROS and subsequent oxidative stress, which results in NASH (Adapted from *Hepatology* 2010, *52*, 774–788) [19].

The current theory of lipotoxicity centres on an increase in the flux of FFAs within hepatocytes (see Figure 1). This is a direct consequence of increased influx (through increased dietary intake of SFAs as well as *de novo* lipogenesis and adipose lipolysis in the setting of insulin resistance and impairment of compensatory oxidative processes [19]. The net result is the generation of toxic lipid metabolites, such as ceramides, diacylglycerols, lysophosphatidyl choline, and oxidised cholesterol metabolites, which act as reactive oxygen species (ROS) [19–21], although the absolute and relative levels of each of these substances in NAFLD remain unconfirmed. Insulin resistance appears to be of central importance in the development and progression of NASH and is critical to the development of oxidative stress and lipotoxicity. A number of genetic and environmental factors appear to interact leading to the development of insulin resistance in patients with NAFLD [22]. Obesity-related adipocyte dysfunction is believed to occur in the setting of increased calorie intake and adipocyte hypertrophy [23] and is characterised by altered levels of adipokines (e.g., adiponectin) [24]. A full exploration of the various metabolic and secretory consequences of increased adipocyte mass in the setting of obesity and insulin resistance is beyond the scope of this review and is better covered elsewhere [25–28]. Furthermore, obesity induces endoplasmic reticulum (ER) stress, which in turn leads to a compensatory response (the “unfolded protein response”) that causes hyper-activation of c-jun terminal kinase (JNK) and further impairment of insulin signalling leading to diabetes mellitus [29].

In animal models, obesity induced by a high-fat diet has also been shown to cause insulin resistance and pro-inflammatory signalling via toll-like receptor TLR4/nuclear factor kappa-light-chain-enhancer of activated B cells (NF-κB) pathways [30]. Chronic hyperinsulinaemia has been shown to further impair skeletal muscle and hepatic insulin signalling in humans, which promotes hepatic steatosis [31]. Insulin-resistant adipose tissue also produces excessive amounts of FFA via lipolysis creating a vicious cycle of accumulating lipotoxic metabolites, steatosis, and insulin resistance [32]. Peripheral adipose tissue also plays a critical role in promoting inflammation and insulin resistance via increased production of pro-inflammatory tumour necrosis factor alpha (TNFα) and interleukin- (IL-) 6 in the setting of obesity [33]. Hyperinsulinaemia and hepatic insulin resistance and steatosis is promoted by increased JNK-1 signalling (via IL-6) in adipose tissue [34] and it is purported that adipose tissue-derived mediators are a major source of damaging cytokines in NASH [35]. Newly-generated FFA, resulting from *de novo* lipogenesis and adipose lipolysis, combine with excess dietary FAs to overwhelm the capacity of protective oxidative metabolic pathways in the liver, skeletal muscle, and the pancreas. Accumulating lipotoxic metabolites, such as ceramides and diacylglycerol, and FFA accumulation in the liver subsequently induces a chronic inflammatory state [19]. This has been shown through *in vivo* murine studies to involve NF-κB activation and is once again characterised by the production of cytokines, such as IL-6, IL-1, and TNFα, resulting in both hepatic and systemic insulin resistance [36]. Mitochondrial ROS, induced by SFAs, appear to contribute to JNK activation and cellular insulin resistance [37]. FFAs have also been shown to activate TLR4 causing apoptosis [38] and the inhibition of TLR4 has been shown to prevent steatohepatitis in mice [39]. Apoptotic pathways are activated by FFAs via the destabilisation of lysosomal membranes causing release of cathepsin B, an activator of apoptosis [40,41]. The relative contribution of each of these and other mechanisms, and their potential as targets for therapeutic intervention in NASH, is the subject of ongoing research. What is clear, however, is that oxidative stress that occurs in the setting of obesity-related insulin resistance and lipotoxicity is central to hepatocyte injury and is critical to the pathogenesis of NASH.

## 4. The Role of Iron in NAFLD and NASH

Hepatic iron is a source of oxidative stress and therefore hepatocyte dysfunction but the role of iron in NAFLD and NASH remains controversial [42]. The first demonstration of a possible pathological effect by George *et al.* from Brisbane, Australia [43] who showed using multivariate analysis that haemochromatosis *HFE* gene mutations were associated with increased hepatic iron, acinar inflammation, and steatosis in 51 patients with NASH. A significant association between the prevalence of *HFE* gene mutations, iron overload and NASH has been variously confirmed and refuted in studies since [44–46]. One possible explanation for these contradictory results is the variable expression of iron loading in different populations.

This question is of practical significance as recent studies have shown that increased hepatic iron concentration in patients with NAFLD is associated with IR [47–49] and therefore may indirectly contribute to disease progression. A recent study from Korea [50] showed that liver diseases that are most commonly associated with IR and diabetes are also associated with iron overload. Furthermore, venesection has been shown to not only lower systemic iron [51] and reduce serum ferritin and hepatic iron concentration but also improve glycaemic control [51–53]. It has been suggested that patients with metabolic disorders, high serum ferritin, and mild-to-moderate iron overload on liver biopsy or MRI represent a distinct clinical entity, termed “insulin-resistance associated hepatic iron overload” (IRHIO), but this is questionable. Most patients in the initial study by Moirand *et al.* [47] proposing this were obese and less than half of the study cohort underwent liver biopsy.

In the steady state, serum ferritin levels reflect total body iron stores and, in the absence of inflammation, raised levels indicate iron overload. In hereditary haemochromatosis, it is well-known that serum ferritin levels are associated with advanced fibrosis [54]. Studies suggest that ferritin can act as a cytokine to induce the release of further tissue cytokines and activate Kupffer and stellate cells, thereby exacerbating fibrosis [55]. In NASH, however, the ferritin level may be elevated out of proportion to liver and body iron stores. Whether ferritin directly (or indirectly as a measure of hepatic iron content) contributes to hepatic inflammation and fibrosis is not clear.

It has been suggested that iron may be involved in the production of oxidative stress on hepatocytes as a traditional “second hit” in the pathogenesis of NASH. Studies have demonstrated a correlation between serum ferritin levels and the presence of NASH [43,54,56,57] and an association between elevated serum ferritin and mild-to-moderate iron overload on multivariate analysis [58]. An important issue, however, is the role of ferritin as an acute phase reactant protein. It has been suggested obesity represents a pro-inflammatory state and levels of inflammatory markers, such as C-reactive protein, are often elevated in diabetes mellitus [59]. This suggests that serum ferritin, as an acute phase reactant, may be a marker of IR rather than hepatic iron overload in NAFLD and NASH [60–62]. Thus, despite evidence of an association, the importance of a raised ferritin in NASH and the role of iron as a source of oxidative stress remain unresolved. Whether a raised ferritin is a surrogate marker of liver inflammation, an indicator of insulin resistance or merely represents a bystander effect of hepatic steatosis is unclear. Further studies are warranted in this area.

## 5. Progressive NASH: From Inflammation to Fibrosis

Hepatic fibrosis is the natural consequence of iterative injury in chronic liver disease that is usually mediated by chronic inflammation [63]. Liver-related complications are related to the progression of fibrosis to cirrhosis in all chronic liver diseases, including NAFLD, and therefore an overview of fibrogenesis in NASH is central to any understanding of pathogenesis. Furthermore, the heterogeneity of fibrosis patterns of fibrosis observed in NASH may provide insights into the factors governing disease progression.

Excessive extra-cellular matrix (ECM), mostly in the form of collagen, is deposited by myofibroblastic cells leading to the development of progressive fibrosis within the liver. The hepatic stellate cell (HSC) is the major myofibroblastic cell in the liver responsible for collagen production and scar formation. These cells are usually quiescent and reside in the sinusoids adjacent to hepatocytes but can become activated in chronic injury and migrate to sites of tissue damage [64]. Once activated, HSCs proliferate and take on a matrix-producing, contractile phenotype, capable of expressing cytokines and chemokines that perpetuate inflammation and fibrogenesis. During HSC activation, perpetuation and proliferation of a fibroblastic phenotype occurs in response to signals from neighbouring cells [65–68], as well as ROS [69] and apoptotic fragments [70] generated from hepatocytes.

Activated stellate cells possess the ability to secrete large amounts of ECM, with collagen-1 being the most important protein and principal component of scar tissue. Production of collagen (and other components of the ECM) is potently stimulated by tissue growth factor-beta (TGFβ) [71]. (Lipid accumulation in hepatocytes has also been shown to induce TGFβ signalling and impair adiponectin activity, supporting a key role for FFAs in the development of hepatic fibrosis) [72]. Other important stimuli of ECM production include lipid peroxidation products [73] and connective tissue growth factor (CTGF) [74]. Myofibroblasts other than HSCs have also been discovered in liver disease and their roles in the development of fibrosis in chronic liver diseases are under investigation. Classical portal fibroblasts [75], activated by monocyte chemoattractant protein- (MCP-) 1 [76], are predominantly involved in biliary fibrosis [77] but may contribute to fibrosis in NASH. Bone marrow-derived myofibroblasts differentiate from pluripotent stem cells and can migrate to the damaged liver [78] but they are currently believed to contribute little to the ECM production in liver injury [79]. They may, however, be important to resolution of fibrosis as an important source of the collagen-degrading matrix metalloproteinases (MMP) 9 and 13 [80]. There also appears to be a circulating population of “fibrocytes” (mononuclear cells with unique haematopoietic cell-surface markers) that can differentiate into alpha-smooth muscle actin (αSMA)-positive and TGFβ-responsive cells with myofibroblastic features [81]. The roles of extra-hepatic myofibroblasts, their interactions with cells within the liver and their ultimate relative contributions to hepatic fibrosis remain unresolved.

A feature of chronic liver diseases is the existence of different patterns of fibrosis according to aetiology. Fibrosis in adults with NASH is classically peri-cellular and forms a dense, reticular network of “chicken wire” fibrosis affecting the lobular liver parenchyma [82]. In paediatric NASH, however, the phenotype is quite different with pure portal fibrosis representing the typical lesion, although the adult phenotype and a mixed pattern can also occur [83–85]. The reasons behind this heterogeneity of fibrosis patterns are unclear but potentially multiple fibrogenic pathways exist in NASH, with progression to an individual phenotype driven by genetic predispositions and complex inter-cellular interactions [85].

Progressive fibrosis in adult NASH is characterised by the development of portal fibrosis that may progress to cirrhosis. Indeed, the absence of portal fibrosis in NASH identifies patients who are extremely unlikely to develop hepatic complications over medium- to long-term follow-up [86]. Observational studies have demonstrated that the presence of portal fibrosis is typically associated with a chronic, portal inflammatory infiltrate [87], as well as a peri-portal DR and expansion of the HPC compartment [88]. More recently an association between portal fibrosis and the DR and increased HPC numbers has also been demonstrated in paediatric NASH [85], suggesting that portal-based factors may be more important to disease progression than traditional lobular factors, such as hepatocyte steatosis and inflammation.

## 6. Cell Death in NASH: A Nexus of Injury and Fibrosis

The mechanisms and cellular reactions that link parenchymal injury with portal fibrosis are currently speculative, although recent studies have provided evidence that the type of cell death in NASH may provide the critical nexus between hepatocyte injury and fibrosis. It has been shown that lipotoxicity leads to cell injury and death, via apoptosis or necrosis, and that this is an important driver of inflammation and fibrosis in liver disease [82,89,90]. Cell death is manifested as hepatocyte ballooning (indicating cellular injury) and the appearance of apoptotic bodies and spotty necrosis (indicating cellular destruction) [91]. Necrosis is characterised by rapid cellular swelling and the lytic release of cellular contents following disruption of organelles and surface membrane integrity [92]. Although this process is a potent pro-inflammatory stimulus, apoptosis probably represents the major mechanism of cell death in NASH that drives inflammation and fibrosis [91].

That apoptosis represents an important nexus linking liver injury and fibrosis [93] is somewhat counter-intuitive. Apoptosis represents a programmed form of cell death involving minimal leakage of cellular contents into the extra-cellular space, which typically minimises the inflammatory response [94]. Increased levels of apoptosis, however, are a feature of NASH. Increased levels of apoptosis are seen in obese patients with NASH compared with controls [95]. In addition, studies have demonstrated increased extrinsic apoptotic pathway activation and increased intrinsic pathway activation [96].

Apoptosis levels also correlate with serum FFA levels [97] and *in vitro* studies suggest that this relationship is dose dependent [98]. Studies have confirmed the importance of hepatocyte apoptosis as a critical downstream effect of lipotoxicity in the setting of excess FFA traffic. Animal and *in vitro* studies have shown that FFAs sensitise hepatocytes to the cytotoxic effects of death ligands (such as tumour necrosis factor-related apoptosis-inducing ligand (TRAIL)) [99,100] and expression of the death receptor Fas (CD95) is higher in liver biopsies with NASH compared with those from patients with simple steatosis or no disease [95,101]. Mice exposed to a high carbohydrate diet and humans with higher circulating levels of FFA also show higher expression of Fas. Murine studies have also demonstrated that FFAs can induce up-regulation of the *p53* tumour suppression gene, expression of the TRAIL-receptor (TRAIL-R) [102] and increased levels of JNK [103–105]—an important regulator of apoptosis that has been shown to promote the development of NASH in mice [106]. Excess FFAs promote mitochondrial dysfunction via the production of ROS [41,107] and sustained JNK activation exacerbates ROS accumulation, which induces hepatocyte apoptosis both directly and indirectly [108–110]. FFAs can also activate the lysosomal pathway redistributing Bax and increasing permeabilisation and cathepsin B release leading to apoptosis [40].

As mentioned earlier, ER stress contributes to disease progression and it has been shown that saturated FFAs directly induce a hepatocyte ER stress response[111], with increased levels of ER stress seen in patients with NAFLD/NASH [112,113]. Prolonged ER stress leads to apoptosis through increased release of calcium from ER and mitochondrial injury, ROS production, induction of pro-apoptotic transcription factors, and increased JNK signaling [114–116]. One study has identified apoB100 as a potential mediator, with up-regulation of apoB100 seen after stimulation of hepatic cells with FFAs leading to increased ER stress [117]. In this study, the presence of ER stress was related to the proportion of saturated to unsaturated FFAs rather than the presence of steatosis itself [118]. This is consistent with another *in vitro* study demonstrating that the addition of a monounsaturated FA attenuates hepatocyte apoptosis and insulin resistance caused by exposure to a SFA, despite causing increased steatosis [119]. These findings further support the notion that disease progression is predominantly mediated by SFA traffic.

Excess apoptosis is pro-fibrogenic in liver disease. Engulfment of apoptotic bodies has been shown to increase HSC activation *in vitro* and leads to the increased production of TGFβ—a potent pro-fibrogenic cytokine[120].

## 7. A Role for Hepatocyte Senescence in Disease Progression

Senescence has been identified in a variety of liver pathologies, including viral hepatitis, NASH and hepatocellular carcinoma (HCC) [121–124], and may be a potential mediator of disease progression in NAFLD. Senescence represents a cellular stress response [125,126] and is an irreversible cell cycle arrest that is probably aimed at limiting the proliferation of critically damaged cells and subsequent tumour development [127]. A number of cellular insults (e.g., oxidative stress due to ROS, ultraviolet (UV) light irradiation, chemotherapeutic agents) can induce senescence [128] and it is characterised by increased expression of cyclin-dependent kinase (CDK) inhibitors, such as p21CIP1 and p16INK4a. The senescent state is also identifiable by the formation of senescence-associated heterochromatin foci (SAHF); e.g., heterochromatin protein-1-beta (HP1β) [129–131] and DNA damage foci (SDF; e.g., gamma-histone 2AX) [132–134] within the nucleus, as well as increased lysosomal beta-galactosidase activity (identifiable using the senescence-associated beta-galactosidase (SA-beta-Gal) assay) [135]. The features of senescence, including types, causes and methods for detection are summarised in Table 1.

Oxidative stress, which is ubiquitous in progressive NASH, appears to be a major driver of hepatocyte senescence [136–139]. Accelerated telomere shortening as an inducer of cellular senescence may occur in the setting of oxidative stress [138,140–145]. It has been shown that cells driven to senescence develop increasing mitochondrial ROS production [146], which may perpetuate the induction of senescence via both autocrine and paracrine pathways.

Senescent cells can mediate disease progression via the active secretion of pro-inflammatory factors that affect the microenvironment, and this represents the adoption of a “senescence-associated secretory phenotype” (SASP) [128]. Senescence, via adoption of a SASP, is therefore postulated to contribute to pathogenesis through the aberrant expression of genes and proteins associated with inflammation and tissue damage, including up-regulation of enzymes that induce ROS and activate NFκB [147]. Although the secretory phenotype of senescent hepatocytes remains unknown, studies in other cell types (such as fibroblasts) have demonstrated that senescent cells secrete a spectrum of proteins that have been previously implicated in the promotion of disease progression in NAFLD, including pro-inflammatory cytokines (e.g., IL-6, IL-8), matrix-degrading enzymes (e.g., MMPs), and other cell signalling molecules and growth factors important to liver disease (e.g., hepatocyte growth factor, platelet-derived growth factor and connective tissue growth factor). A core panel of secreted proteins appears to be generally conserved across different cell types once senescent, although the quantity and spectrum of secreted proteins may be affected by the type of injury and the cell involved [128,148–150]. Using a novel *in vitro* model of senescence in cultured HepG2 cells [151], a preliminary characterisation of the hepatocyte SASP *in vitro* is being undertaken by the author (R.S.) although the SASP of primary hepatocytes or hepatocytes *in vivo* has not been studied to date [119].

Although the hepatocyte SASP has not been definitively characterised, there is ample evidence that typical SASP products play a role in the progression of chronic liver diseases, like NASH. It is well-established that chemotactic factors that recruit inflammatory cells to the site of injury represent an important component of the SASP [128]. Neutrophils have been shown to co-localise with senescent cells in response to IL-8 [152], and a number of senescence-associated chemokines (growth related oncogene (Gro)-α, MCP-1, and IL-8) are chemotactic for inflammatory cells in liver disease [134]. Other SASP factors, such as CXC chemokine ligand (CXCL) 16 and CX3C chemokine receptor (CX3CR) 1, are expressed by epithelial cells to attract T cells and other inflammatory cells to the site of injury [153–155]. A recent study investigating the role of senescence in primary biliary cirrhosis demonstrated that senescent biliary epithelial cells (BEC) up-regulate the expression of several chemokines, including CC chemokine ligand (CCL) 2 and CX3C chemokine ligand (CX3CL) 1, that are chemotactic for monocytes and T cells [156]. Some cytokines produced by senescent cells (e.g., IL-6, IL-12, and TNFα) are also capable of antigen-independent bystander T cell activation [157,158] while B cells probably differentiate in response to the profile of senescence-associated factors secreted within the microenvironment, and in turn shape the local inflammatory response [157].

Increased senescence does appear to be associated with progression of disease in NASH. A study comparing liver biopsies from normal controls and patients with NAFLD found that hepatocyte p21 expression was significantly associated with stage of fibrosis and clinical outcomes, such as the development of hepatocellular carcinoma, the need for liver transplantation and liver-related death [123]. Examination of paired biopsies in that study found that features of senescence (increased nuclear size, increased p21 expression) were also strongly associated with changes in fibrosis stage over time. Further associations were demonstrated between improvements in fibrosis and reductions in p21 expression, while worsening fibrosis was associated with increased nuclear size. Similar findings have also been demonstrated in alcoholic liver disease, the histopathology of which is virtually indistinguishable from NAFLD, with increased p21 expression being strongly associated with fibrosis stage and adverse clinical outcomes [159]. In that study, it was also demonstrated that areas of increased p21 expression appeared to correlate with increased αSMA expression, suggesting a link between hepatocyte senescence and HSC activation and ensuing fibrosis.

Senescence may also have a role in limiting fibrosis and/or stimulating regeneration in liver disease. In a study of carbon tetrachloride (CCl_4_) induced liver injury in mice, initial activation of HSCs was followed by the adoption of a senescent HSC phenotype with associated up-regulation of MMPs and down-regulation of ECM components, thereby facilitating reversion of fibrosis [160]. In the same study, disabling the senescence machinery (via knockout of p53) attenuated this effect and *p53*^−/−^ mice developed increased fibrosis. In another mouse model (of fatty liver disease), mature hepatocytes expressed markers of senescence and this was associated with an expansion of the progenitor cell population compared with control animals [161]. This was accompanied by signs of oxidative damage and was seen even where liver damage was subtle suggesting that senescence may be important in early disease. In a study of human specimens from controls and patients with NAFLD, increased HPC expansion was seen in association with increased stage of disease, including those with steatosis alone [88]. Furthermore, the degree of associated DR was strongly associated with the extent of replicative arrest (by p21 staining) after multivariate analysis, suggesting that the DR may be induced in the setting of an increased senescence burden and play a mediating role in the progression of portal fibrosis. The roles of senescence and the hepatocyte SASP in the progression (or regression) of fibrosis in NASH warrants further investigation.

## 8. The Hepatic Inflammatory Response: Implications for NASH

The innate immune response is an important effector of parenchymal inflammation in liver diseases, such as NASH, and is mediated by innate immune cells, including neutrophils, macrophages (Kupffer cells), natural killer (NK) cells, and natural killer T (NKT) cells. The importance of the liver as an organ of the innate immune system is evidenced by the fact that the vast majority (80%–90%) of fixed, tissue macrophages are, in fact, Kupffer cells [162].

Macrophages closely approximate with collagen-producing HSCs [163] and can directly activate stellate cells via the release of IL-6 and TGFβ [157,164], although a number of potential macrophage-derived factors have been described whose expression correlates with NASH severity or HSC activation (e.g., chitotriosidase) [22,165,166]. They are also capable of promoting HSC apoptosis (via TRAIL) [167] and matrix degradation (via MMP-13 and MMP-9) [168,169], and act as an important source of the pro-inflammatory cytokines TNFα and IL-12 [162]. Neutrophils are part of the initial inflammatory response to injury, probably most importantly mediated via the IL-1-receptor [170] and a recent study identified that patients with NASH have elevated peripheral neutrophil-to-leukocyte ratios compared to those without NASH [171]. NK cells are abundant in the liver and represent an important innate defence mechanism against viruses, via the secretion of cytokines that repress viral replication and initiate an immune response. They may have a role in the progression of NAFLD as they are capable of inducing apoptosis of both HSCs and hepatocytes, via IFNγ [162]. Although circulating NK cell numbers and cytotoxic activity appear to be reduced in obesity [172], it is likely that the hepatic NK cell population is significantly increased in NASH [173]. NKT cells regulate host responses to tissue damage by inducing an adaptive immune response (both Type 1 and Type 2) and are seen in association with necroinflammation [174]. Although animal models and studies in other chronic liver diseases have shown conflicting roles in hepatic fibrogenesis [175–177], NKT cells are activated by lipid antigens and therefore may contribute to the progression of fibrosis in NAFLD. Studies in humans and animals suggest that NKT cells may play a protective role in limiting steatosis but a deleterious role in the progression of inflammation and fibrosis [178–181].

The adaptive immune response also appears to play a role in the progression of fibrosis. Animal studies have shown that depletion of T cells is associated with reduced fibrosis [175,182]. The mechanisms are unclear but CD4^+^ T cells likely interact with fibroblasts and macrophages, whereas CD8^+^ T cells appear to increase HSC activation both directly [182] and indirectly by amplifying injury via a bystander effect driven by the local cytokine milieu [157]. The importance of B cells has been highlighted by a recent study showing that mice deficient in B cells show a reduction in fibrosis after hepatocyte injury [183], apparently independent of antigen presentation. B cells have also been shown to secrete pro-fibrotic cytokines, including IL-4, IL-6, and IL-13, and their survival in fibrotic tissue is enhanced by other cytokines (e.g., IL-2) that are generated as part of the immune response [184,185]. The adaptive immune response is traditionally characterised according to the predominant type of T-helper cell subset that predominates, promoting either as a pro-inflammatory Th1 or pro-fibrotic Th2 immune response. CD4^+^ T-helper cells also include regulatory T cells (Treg) and a third distinctive immune response, the Th17 response, has also been identified [186]. The role of the adaptive immune response in the pathogenesis of NASH is discussed in more detail below.

Stellate cells also participate in the innate immune response by expressing a range of chemokines that actively recruit leukocytes, and activated HSCs continue to do this in the absence of other pro-inflammatory cytokines [157]. Animal studies with targeted disruptions of chemokines responsible for lymphocyte recruitment show reduced fibrosis [187]. The transition from an acute injury to a chronic inflammatory state is in part mediated by these factors secreted within the microenvironment that facilitate close interactions between leukocytes and fibroblasts [188]. Cytokines secreted by T cells are important activators of HSCs [157], while activated HSCs are themselves able to act as antigen-presenting cells to stimulate T cells (including NKT cells, CD4^+^, and CD8^+^ T cells) [189]. In chronic injury, it is believed that these interactions promote a stromal milieu that facilitates the inflammatory response and causes a dysregulated tissue repair response, resulting in fibrosis [190].

In short, the inflammatory response that occurs in NASH involves both the innate and adaptive immune systems. This cascade begins with hepatocyte injury in the setting of insulin resistance and lipotoxicity and is propagated by cellular apoptosis (and potentially hepatocyte senescence), culminating with the activation of HSCs and ensuing fibrosis. The intra-cellular mechanisms that are involved in the propagation of inflammation and fibrosis will be examined next.

## 9. Sterile Inflammation, Toll-Like Receptors and Propagation of the Inflammatory Response

The propagation of inflammation and ensuing fibrosis in parenchymal liver diseases usually occurs as the result of antigenic exposure and an appropriate inflammatory response. No recognised antigen exists in NASH although studies have clearly demonstrated the contribution of both the innate immune system and a chronic, cell-mediated inflammatory response. As previously discussed, excess FFAs and oxidative stress represent the primary drivers of this inflammatory response, principally via the promotion of hepatocyte injury (causing apoptosis and/or necrosis) and the induction of hepatocyte senescence. Ongoing research is now focused on these mechanisms and the inter-cellular interactions and signals that propagate inflammation and provide a link with fibrogenesis. It is appropriate to point out that the metabolic dysregulation associated with obesity and insulin resistance that promotes the chronic inflammatory state resulting in hepatic fibrogenesis also contributes to dysfunction of other organs, although extra-hepatic consequences are beyond the scope of the current review.

An immune response in the absence of pathogens is termed “sterile inflammation” and there is evidence that sterile inflammation plays an important role in diseases of the liver [191–193]. This inflammation is characterised by the release of damage associated molecular patterns (DAMPS), which are molecules that are liberated by injured cells capable of triggering inflammation [194,195]. Indeed, the cellular interactions involved in the DAMP-associated immune response mirror those that mediate an inflammatory response in the presence of pathogens—via equivalent microbial pathogen associated molecular patterns (PAMPs) [196,197]. Once DAMPS are released, cytosolic machinery known as the “inflammasome” is up-regulated to promote production of the canonical pro-inflammatory cytokine IL-1β [198]. Although DAMPS are most commonly released during necrotic cell death rather than apoptosis [199], apoptotic cells that are not removed by phagocytes can act as a source of DAMPs as they undergo a secondary necrosis [200]. Release of DAMPs can also be induced via mechanisms independent of apoptosis (e.g., in response to oxidative stress with the release of macrophage-derived high mobility group box 1, HMGB1) [201,202].

TLRs are pattern recognition receptors for PAMPS and DAMPS that induce signalling pathways that regulate pro-inflammatory cytokines and chemokines. TLRs play an important role in the initiation and progression of hepatic inflammation and therefore represent conspicuous candidates as mediators of disease progression in diseases such as NASH. TLRs are expressed in a variety of liver cells. Kupffer cells express TLR2, 3, 4, and 9 and these receptors are responsive to multiple triggers [203]. When stimulated with lipopolysaccharide (LPS), Kupffer cells play a predominant role in mediating cytokine release [204] and produce chemokines and inflammatory cytokines (such as TNFα, IL-1β, IL-6, IL-10, IL-12, and IL-18) [205]. Studies have shown that Kupffer cells act to stimulate fibrogenic responses via production of TGFβ1, MMPs, platelet-derived growth factor, and ROS [206]. In mice, depletion of Kupffer cells using clodronate significantly reduces the severity of NASH [207]. TLRs are also expressed by hepatocytes [208], HSCs (which express TLR2, 4 and 9 on activation) [209,210], sinusoidal [211,212] and biliary [213] epithelial cells, as well as hepatic dendritic cells [214].

There are many different kinds of DAMPS. Mammalian DNA itself, just like bacterial DNA, can act as a DAMP via the activation of TLR9 and this mechanism of pathogenesis has been demonstrated in diseases of acute parenchymal injury, such as pancreatitis and paracetamol toxicity [215–221]. Mitochondria are a rich source of DAMPS [221,222] and their signalling molecules, such as ATP and formulated peptides (e.g., encoded by the NLRP3 gene), can induce inflammasome activation [221,223,224]. Mice with constitutively active NLRP3 have an overactive inflammasome pathway, which induces liver injury [225]. Additionally, increased ATP levels can propagate inflammation directly (via the P2X7 receptor in the inflammasome leading to increased IL-1β production) [226] and indirectly (via promotion of bacterial product entry into cells leading to activation of PAMPs) [227]. HMGB1 is a nuclear protein that has also been recognised as a DAMP, secreted by injured or necrotic parenchymal cells or by immune cells in response to other PAMPs and DAMPs [228–232].

Kupffer cells are believed to play a key role in sterile inflammation and studies have confirmed the role of macrophages as IL-1 secreting sentinels in the immune response [195,233,234]. Kupffer cells sense DAMPS and are responsible for neutrophil recruitment. In mice, CD11b-positive Kupffer cells (which predominantly secrete pro-inflammatory factors rather than phagocytose debris) produce IL-1α and initiate the neutrophil response when necrotic cells are instilled in the intraperitoneum [235]. Furthermore, mice with depleted Kupffer cells (via clodronate administration) show reduced levels of caspase, IL-1 and neutrophil recruitment, similar to those with impaired P2X7 receptor signaling [234].

The vascular endothelium also functions as a sentinel network to detect DAMPS. Endothelial cells have receptors for multiple DAMPS [236–238] and respond to TLR9 secretion from necrotic hepatocytes, resulting in the up-regulation of IL-1β and subsequent neutrophil-mediated inflammation and parenchymal damage [215]. In the liver, neutrophils adhere within the sinusoids rather than the post-capillary venules via selectin-independent mechanisms, such as the ATP-activated P2X7 receptor and NLPR3 inflammasome [234,239,240]. Various stromal cells, such as epithelial, mesenchymal and mesothelial cells, have also been shown to act as sentinels for DAMPS.

Studies suggest that TLRs are essential to NASH pathogenesis [207,241–245]. The key mediators of sterile inflammation, namely TLR4 and TLR9, appear to be directly linked to hepatocyte apoptosis, alanine transaminase (ALT) levels and fibrogenesis, and these parameters are reduced in the absence of TLR4 and TLR9 [39,207,241]. TLR4 is of particular interest in NASH as it is a potent activator of innate immunity when stimulated by bacterial LPS [246]. This occurs through activation of MyD88 and TRIF pathways, triggering the initial activation of NF-kB and MAPK [247]. TLR4-deficient mice have lower levels of liver injury and lipid accumulation when compared with wild-type mice following a methionine/choline-deficient (MCD) or high-fructose diet [39,207]. Elevated levels of TLR4 ligands (such as LPS) have been demonstrated in animal models of NASH [248] and increased levels of LPS in portal blood are seen in association with NAFLD [249]. Prospective murine models have demonstrated that infusion of LPS results in weight gain, insulin resistance, and increased liver triglycerides [250], while decreased levels of LPS in mice correlate with reduced steatosis and fibrosis [250–254]. Disrupted intestinal epithelial function can occur in the setting of chronic liver disease and there is currently great interest in the contribution of increased amounts of bacterial products from the gut, especially LPS, to the disease progression in NASH [255–257]. It has also been suggested that bacterial and endotoxin translocation may be mediated via TLR2. *TLR2*^−^/^−^ knock-out mice, using a bile duct ligation model of liver injury, showed lower levels of translocated bacteria and bacterial products and reduced fibrosis [258].

TLRs have been shown to recognise FFAs and denatured host DNA [259–261], however it is unclear whether the FFAs stimulate TLR4 directly or do so through their association with increased levels of LPS [262]. HMGB-1 is also recognised by TLR4 [263] and has been linked with severity of liver injury in mouse models [264]. TLR9 has also been studied extensively and it is needed for Kupffer cells to produce IL-1β, which induces hepatocyte death. TLR9-deficient mice are protected from liver steatosis, inflammation, injury and fibrosis induced by a choline-deficient diet when compared with wild-type animals [241], and are protected from injury by CCl_4_ or acetaminophen [215,259].

Recent studies have established a link between NASH and increased IL-1β production [241,264–268], potentially via an increase in inflammasome activation [264]. Activation of the inflammasome and release of IL-1β requires 2 signals: an endogenous danger signal and a TLR ligand [269]. A recent study has demonstrated that SFAs can elicit inflammasome activation [270]. SFAs are known to increase sensitivity of primary hepatocytes to LPS *in vitro*, and administration of exogenous LPS *in vivo* can enhance IL-1β levels and inflammasome activation in the setting of steatohepatitis. Evidence that SFAs induce hepatocyte apoptosis [114,119,271] and DAMPS from dying hepatocytes can induce inflammasome activation and IL-1β [264], further suggests that SFAs “prime” the fatty liver for LPS-induced inflammasome activation [264].

Fibrogenesis in NASH may be linked to TLRs as well. Elevated levels of LPS are found in models of hepatic fibrosis [272–274] and in patients with cirrhosis [275,276]. TLR signalling activates NF-κB, which up-regulates the profibrogenic TGFβ signalling pathway [272]. The role of TLR9 has been demonstrated in murine models with *TLR9*-knockout mice showing less fibrosis (and less severe hepatitis) than wild type mice. A reduction in fibrosis appears to be associated with a reduction in IL-1β as well [241], supporting its critical role in disease progression. Kupffer cells are the major source of IL-1β in NASH and they act to promote increased lipid accumulation in cultured hepatocytes [241,277,278], sensitise hepatocytes to injury [279,280], and directly activate HSCs inducing liver fibrosis [241].

Another signalling pathway that has attracted considerable interest is the hedgehog (Hh) pathway, which is important in cellular proliferation and differentiation and plays a role in tissue repair and potentially fibrogenesis. Recent studies, most notably by Diehl and colleagues, have demonstrated that Hh ligand expression strongly correlates with hepatocyte injury and Hh activity induces the activation of HSCs and recruitment of inflammatory cells [178,281,282]. Furthermore, it has been demonstrated that Hh stimulates the production of CXCL16 from immature ductular epithelium, with resultant infiltration of NKT cells capable of promoting a Th1 or Th2 response, and is associated with HPC activity [283]. Elegant murine models of steatohepatitis have investigated the potential role of Hh and osteopontin, a pro-inflammatory cytokine that is an Hh target, showing differing levels of fibrosis in knockout mice with altered Hh or osteopontin activity [178,284]. These results are supported by findings in humans with steatohepatitis [178,179] and it is hypothesised that aberrant Hh expression may explain the heterogeneity of fibrosis seen in NASH.

## 10. The Immune Response in NASH: A Th17 Response?

Although the liver is enriched with cells of the innate immune system [285], the adaptive immune system is also important, as evidenced by an association between a chronic inflammatory infiltrate affecting the portal tracts in NASH and a more progressive phenotype of significant fibrosis [87,286]. A preliminary characterisation of the portal inflammatory infiltrate in 33 patients with NAFLD by the senior author (R.S.) and colleagues demonstrates that it comprises predominantly CD8^+^ T cells and CD68^+^ macrophages, with smaller numbers of CD4^+^ T cells, CD20^+^ B cells, and CD56^+^ NK cells [287]. These observations support a role for the adaptive immune response in advanced NAFLD, although significant increases in the numbers of portal CD68^+^ macrophages were also seen in patients with simple steatosis alone in association with increased expression of CCL2. This suggests that macrophages play a critical role in early disease and are consistent with previous studies highlighting their importance in disease progression [288,289].

Leukocyte infiltration begins with adhesion to the endothelium followed by migration into the parenchyma [290,291]. Potential sources of recruiting chemokines include nearby Kupffer cells, hepatocytes, other leukocytes and activated HSCs [157]. The passage of leukocytes through the liver is complicated by its unique microvascular anatomy. Following acute injury, leukocytes enter via the sinusoids in response to cytokines (e.g., IL-1, TNFα, and IFNγ) and chemokines (e.g., CXCL9 and CXCL10) [292,293]. They subsequently migrate via the space of Disse to the portal tracts and on to the lymph nodes [294,295]. This may explain how cells of the immune response could be “activated” within the hepatic parenchyma via signals and factors expressed in response to *lobular* injury before congregating as a *portal* inflammatory infiltrate capable of directing the progression of disease.

Naive CD4^+^ T cells differentiate into effector T-helper cells in response to cytokines produced by the innate immune system. Discovery of two divergent pathways of T cell differentiation (the Th1 and Th2 responses) has led to a greater understanding of the pathogenesis of disease in many organ systems [296]. The Th1 response is central to the clearance of intracellular pathogens and is initiated by IFNγ and IL-12. The Th2 response, on the other hand, is activated in response to extracellular pathogens via IL-4. This has led to the concept that these two pathways involve a mutually exclusive terminal differentiation of T cells in response to chronic stimulation [297]. The type of immune response is critical to the development of fibrosis. The Th1 response typically generates a prominent inflammatory reaction but little fibrosis, whereas the Th2 response is more fibrogenic. Studies have shown that the Th2 response, particularly the expression of IL-13, is associated with the transcription of many genes integral to fibrogenesis, including procollagen I and III, MMP-2, MMP-9, and tissue inhibitors of metalloproteinases (TIMPs) [298]. Furthermore, blockade of the classic Th2 cytokine IL-13 is associated with reduced hepatic fibrosis [299].

Analysis of the immunohistochemical and gene expression profiles of liver specimens from patients with a spectrum of NAFLD suggests that progressive fibrosis in NAFLD is predominantly supported by a Th1 immune response. Patients with NASH (*versus* simple steatosis alone) show significantly increased expression of IL-1β and TNFα, while increased IL-6 expression is seen in patients with portal fibrosis (versus lobular fibrosis) [287]. A predominant Th1 immune response in the setting of progressive fibrosis is somewhat counter-intuitive. The Th2 response is traditionally regarded as pro-fibrogenic with collagen synthesis stimulated by IL-4 and IL-13 and pro-inflammatory cytokine production and neutrophil chemotaxis down-regulated by IL-10, its other key effector cytokine [182]. Future studies using micro-dissection techniques to examine location-specific gene expression in association with different stages of disease and/or distribution of fibrosis (*i.e*., portal *versus* centrilobular) are warranted.

Recently, another subset of effector Th cells has been identified that secretes IL-17 and exhibits functions shared by both the Th1 and Th2 responses [300]. These cells have been labelled Th17 cells and arise when pathogens are not adequately cleared by either response, thereby representing a link between the innate and adaptive immune responses. Th17 cells are potent inducers of inflammation, predominantly mediated by neutrophils (which are the principal cellular target of IL-17) [301]. Their involvement has been demonstrated in a number of human autoimmune and inflammatory conditions (such as rheumatoid arthritis and inflammatory bowel disease) [302,303]. The full functional capacity of Th17 cells is still being elucidated, but they principally mediate inflammation, vessel activation and matrix re-modelling through the effect of IL-17 on neutrophils, dendritic cells and macrophages, endothelial cells, and fibroblasts, respectively [301]. Little is known about the regulation and termination of the Th17 response, but the combination of TGFβ and IL-6 has been shown to promote the induction of Th17 T cells from naïve T cells, synergistically stimulate the production of large amounts of IL-17 [304], and. inhibit the generation of Foxp3^+^ regulatory T cells, which normally protect against tissue injury by suppressing the inflammatory response [305]. *In vitro* and *in vivo* data suggest that IL-6 is the predominant Th17-inducing cytokine [306]. Th17 cells play a key role in leukocyte recruitment, namely neutrophils, by secretion of IL-17A, IL-17F, IL-21, IL-22, and TNFα [307,308]. IL-17 activates and mobilises neutrophils both directly and through chemokines CXCL1, CXCL2, and CXCL8 [309,310]. IL-17 is also known to act on hepatocytes to increase expression of inflammation-associated genes, chemokines and CRP, and this action is enhanced by the addition of TNFβ or IL-1 [311,312].

The Th17 response has been implicated in a number of autoimmune diseases such as inflammatory bowel disease, rheumatoid arthritis and multiple sclerosis [313–315]. Currently, the specific contribution of a Th17 response to the progression of NAFLD is incompletely understood, although IL-6 and TGFβ are known to be over-expressed in NASH and could induce a Th17 response [287,316]. Alcoholic liver disease has a near identical histological appearance compared with NAFLD and increased plasma levels of IL-17 and liver infiltration with IL-17-positive cells, correlating with fibrosis severity have been described in both aetiologies [287,317,318]. Furthermore, in alcoholic liver disease, HSCs have been found to express IL-17-receptors and recruit neutrophils in response to IL-17, suggesting a direct role in the progression of cellular injury. IL-17 appears to exacerbate FFA-induced hepatocyte steatosis and neutralisation of IL-17 attenuates LPS-induced liver injury in mice on a high fat diet [318].

It has been suggested that the presence of a periportal and perivenular neutrophil infiltrate and increased levels of IL-6 in NASH support a functional role for the Th17 response in mediating progression of NAFLD [186]. At this stage, experimental evidence can only suggest a “functional correlation” between inflammation induced by lipotoxicity and cellular injury in the setting of oxidative stress and the Th17 response [186]. In a study of patients with NAFLD, the presence of IL-17-positive cells was also associated with the presence of portal inflammation and degree of DR [287], suggesting that the Th17 response may be an important mediator of portal-based mechanisms that have been implicated in fibrogenesis [85,88]. Furthermore, previous studies have demonstrated that IL-17 stimulation of BEC induces production of IL-1β and IL-6 [319]. These cytokines are capable of sustaining the Th17 response in humans [186] and provide a potential nexus between an evolving DR and disease progression.

**Figure 2.**
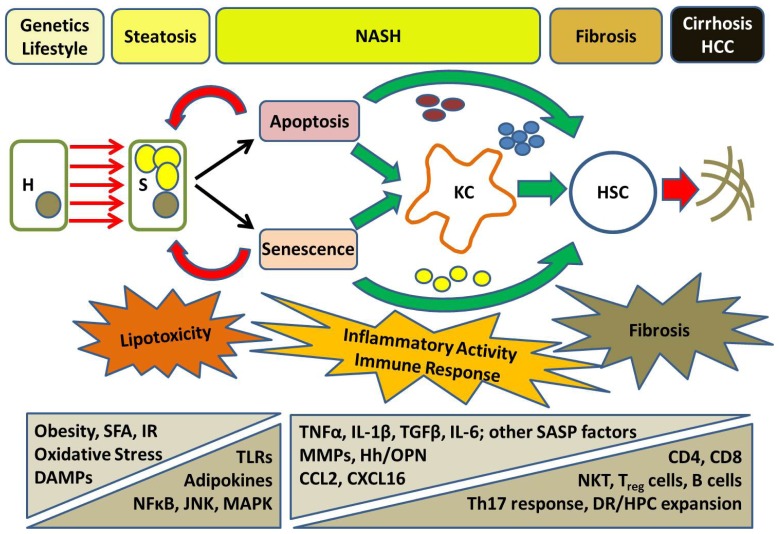
A schematic conceptualising the pathogenesis of NASH. Hepatocytes (H) are affected by lifestyle factors (a diet high in saturated fatty acids (SFA), obesity) and genetic predispositions contributing to the development of insulin resistance (IR) and hepatic steatosis (S). In some patients, these multiple parallel metabolic hits lead to cellular damage, via a process called “lipotoxicity”, involving excessive oxidative stress principally driven by the lipotoxic metabolites of SFA. Injured hepatocytes release DAMPs that initiate an inflammatory response, predominantly via toll-like receptors (TLRs), and activate pro-inflammatory signalling pathways in the setting of increased adipokine levels. Although injured hepatocytes undergo necrosis, apoptosis and senescence are alternative cell fates that are likely to be of greater importance to disease progression. Direct recruitment of Kupffer cells (KC) and other components of the innate immune response occurs with activation of the inflammasome and the coordinated release of pro-inflammatory and pro-fibrogenic cytokines and ligands (e.g., Hedgehog; Hh, and osteopontin; OPN). Hepatic stellate cells (HSC) are subsequently activated to produce extra-cellular matrix leading to progressive fibrosis, cirrhosis and its complications (e.g., hepatocellular carcinoma; HCC). Engulfment of apoptotic bodies and factors produced by senescent cells (upon adopting a “senescence-associated secretory phenotype; SASP”) can also influence HSC activity directly. The activity of KC promotes a pro-inflammatory microenvironment that initiates an adaptive immune response, likely representing a Th17 response. The appearance of a chronic portal inflammatory infiltrate accompanies a ductular reaction (DR) and hepatic progenitor cell (HPC) expansion. These factors are associated with progressive fibrosis that likely represents an imbalance of tissue damage and repair due to the influence of different inflammatory cells.

An evolving appreciation of the role of the immune system and interactions between different cell types has led to a new conceptualisation of the pathogenesis of NASH (summarised in Figure 2). Further work is required to fully elucidate the details of key interactions and mechanisms that stimulate and mediate fibrogenesis but this model provides a useful overview of the progression of disease from steatosis and inflammation to fibrosis and cirrhosis.

## 11. Treatment of NASH: Current Therapies and Potential Targets

It is generally regarded that effective treatments in NAFLD are limited [320,321]. One explanation for this is the fact that the pathogenesis of NASH, as discussed above, clearly involves the complex interaction of cellular responses to chronic injury. Furthermore, the development of a dysfunctional metabolic milieu over many years will likely not be easily repaired with short-term intervention and many studies to date have involved short-term treatment only. In addition, there is an increasing appreciation that NAFLD is probably a heterogeneous disease. Conflicting results in therapeutic trials to date may reflect the fact that treatment cohorts are made up of patients with differing genetic and immunological responses to the same metabolic problems (e.g., obesity, insulin resistance, unhealthy diets high in calories and saturated fats) and are therefore unlikely to respond to interventions in the same way. The pathogenesis of NASH involves multiple parallel metabolic hits and potentially multiple fibrogenic pathways are involved. An increasing understanding of the pathogenesis of NAFLD will likely improve our development and use of interventions in this disease. To date, some interventions demonstrate reduction in disease activity but few therapies are associated with improvements in fibrosis scores and generally larger studies with longer follow-up periods are needed [321]. It is prudent, however, to provide an overview of therapeutics in this area and examine how our understanding of pathogenesis has, and will continue to, inform our development of treatments for NAFLD.

Early models of disease pathogenesis held that the development of steatosis was a necessary first hit preceding the development of inflammation and ensuing fibrosis. It seems reasonable to assume then that therapies that reduce the burden of steatosis would be effective in limiting fibrosis. Indeed, a mainstay of current treatment for NAFLD remains lifestyle changes aimed at weight loss through diet and exercise. A meta-analysis of randomised controlled trials of diet and exercise shows a consistent benefit for patients in terms of improvements in transaminase levels and some histological parameters (e.g., steatosis, hepatocyte ballooning, necroinflammtion) [321]. The degree of weight loss may be important with mild weight loss (~5% of body weight) improving steatosis and greater weight loss (~7%–10% of body weight) improving inflammation [322,323], although these results are unconfirmed [324]. Alternatives to weight loss in combating steatosis include the use of blood orange juice, which induces the expression of peroxisome proliferator-activated receptor-a and lipid oxidation via increased acylCoA-oxidase production, although initial experiments in mice remain unvalidated [325].

Lifestyle interventions are safe and also generally improve insulin resistance [321], which is believed to be an important driver of pathogenesis, although this benefit may depend on carbohydrate restriction rather than caloric restriction alone. Bariatric surgery (laparoscopic gastric banding, Roux-en-Y approaches, gastric sleeve operations) has enjoyed excellent results in terms of weight loss and reversal of insulin resistance in obese patients but its role in the management of NASH is controversial [324]. Although it appears that bariatric surgery improves steatohepatitis on liver biopsy [326], studies examining its effect on fibrosis are conflicting [327–329] and its impact on the natural history of NAFLD remains unresolved. It seems reasonable, therefore, to conclude that interventions aimed at reducing steatosis may be capable of also modulating associated pathogenic mechanisms (*i.e*., insulin resistance, necroinflammation) but may not be enough to limit the progression of fibrosis, necessitating a search beyond steatosis and inflammation.

The lipotoxicity model in NASH attributes a central role to oxidative stress in the progression of disease and therefore the use of antioxidants and other approaches aimed at limiting oxidative stress have attracted considerable attention. The agent with the most evidence is vitamin E with a large multi-centre, randomised controlled trial demonstrating improvements in all histological parameters, except fibrosis scores, in patients treated with 800 IU/day *versus* placebo for 96 weeks in patients without diabetes [330]. Tempering the enthusiasm for the use of vitamin E in NAFLD is the suggestion that the use of high-dose vitamin E is associated with an increase in all-cause mortality [331,332], although the data are inconsistent and more recent analyses question this association [333,334]. It is commonly believed that it is the antioxidant properties of vitamin E that confer any benefit in the treatment of NASH. This may be an overly simplistic appraisal of the actions of vitamin E and experts point to differing cellular effect of vitamin E analogues despite identical antioxidant properties [335]. In support of this is the fact that other agents with antioxidant properties (e.g., *N*-acetylcysteine, vitamin C, probucol, betaine) have not been shown to be effective in treating NASH [321,336].

More recently, a negative correlation has been demonstrated between the degree of coffee consumption and fibrosis stage in NASH [337]. Interestingly, this effect appears to be independent of caffeine suggesting that another of the more than 1000 compounds found in coffee is responsible [336]. Once again, it remains unclear whether these results are due to the antioxidant properties of coffee. Other dietary factors that warrant further investigation are the use of probiotics, aimed at maintaining intestinal microbiota homeostasis, as well as l-carnitine, which regulates fatty acid turnover and limits oxidative stress [338,339].

The role of phlebotomy is somewhat controversial, although if significant iron overload influences the progression of hepatic fibrosis by promoting oxidative stress then depletion of iron should at least attenuate this. In a study of 587 Italian patients with NAFLD (compared with 187 healthy controls), Valenti *et al.* demonstrated that hepatic fibrosis was more common in patients with stainable iron within hepatocytes. Phlebotomy has been demonstrated to be a safe and effective treatment to combat the detrimental effects of iron overload in patients with NAFLD [340]. In this study, serum markers of iron overload and liver injury in 32 patients with NAFLD significantly improved after phlebotomy. Other studies have also shown blood-letting to be beneficial in improving IR, which may have a direct impact on disease progression in NASH [51,53,341,342]. More recently, a study of 53 patients with NAFLD (abstract only) did not show that phlebotomy was useful for improving hepatic steatosis, liver enzymes, or insulin resistance, although these patients did not undergo liver biopsy to confirm the distribution of iron [343]. Based on current evidence and its safety profile, phlebotomy may still represent a reasonable treatment option for patients with significant fibrosis where iron accumulation is shown to be predominantly hepatocellular.

Newer approaches to treatment of NASH are aimed at the myriad of inter-cellular mechanisms that have been implicated in the pathogenesis of NASH. Pentoxifylline is a cytoprotective agent that inhibits the pro-inflammatory cytokine TNFα. The best quality data for its use in NAFLD comes from a randomised controlled trial of 55 patients with NASH treated with pentoxifylline or placebo for one year. Treated patients showed significant improvements in inflammation and steatosis, without increased adverse effects [344]. Reduction in fibrosis was not statistically significant although the study may have been under-powered. Interestingly, a follow up study analysing patients’ plasma before and after treatment showed a significant reduction in oxidised FAs in treated patients suggesting that the beneficial effects may (in part) be due to depletion of free radicals capable of inducing oxidative stress [345]. A study of the anti-TNFα agent, infliximab, in a dietary model of steatohepatitis in rats showed improvements in liver enzymes and histological parameters, including fibrosis [346], although hepatic outcomes in humans treated with infliximab to date remain unclear. Other proof of concept studies demonstrating improvements in histological, biochemical and/or inflammatory parameters in animal models of steatohepatitis and fibrosis that warrant further exploration include the use of JNK-inhibitors [347,348], STAT3 blockade [349], and anti-CD3 therapy [350,351]. In the case of anti-CD3 therapy, a phase I study testing the safety of an oral monoclonal anti-CD3 antibody demonstrated reductions in IL-1 and IL-17 expression and induction of regulatory T-cells without increased adverse effects [352]. A follow-up phase 2a study of the agent in NASH patients found reductions in biochemical parameters of inflammation and insulin resistance, as well as induction of regulatory T-cells, without changes in circulating CD3^−^, CD4^−^ or CD8^−^ positive cells, after treatment [353,354]. Unfortunately, these preliminary results have not been formally published or replicated to date. A number of TLR inhibitors have been developed for other indications (e.g., OPN305 and E5564 for sepsis, OPN401 and N10101 for inflammation, and IRS954 for auto-immune disease) and it seems prudent to re-visit these as therapeutic options as the importance of TLR-mediated injury in NASH grows. Reducing LPS influx from the gut has been investigated as a therapeutic target. A phase II open-label trial of hyperimmune bovine colostrum, which neutralises LPS, demonstrated improvements in markers of insulin resistance and liver enzyme levels in patients with biopsy-proven NASH [355]. Although effects on histology were not studied, treatment was associated with an increase in Foxp3^+^ cells reinforcing the importance of enhanced regulatory T-cell activity as a potential therapeutic target. Additionally, reduced murine liver damage due to alcoholic steatohepatitis was observed after blockade of the IL-1 receptor and subsequent reduction in inflammasome-dependent signaling [356]. Whether this could be translated to NASH, however, is uncertain as some inflammasome signalling is stimulus specific and alternative, caspase-1-independent pathways of IL-1β exist. Nevertheless, guided by a growing understanding of the pathogenesis of NAFLD, immunomodulatory therapy as a treatment modality NASH warrants further exploration.

Finally, patients with the metabolic syndrome are at increased risk of liver disease due to NAFLD and a dysfunctional metabolic milieu, particularly in the setting of obesity, has been implicated in the pathogenesis of NASH. The use of specific medical therapies that are effective in patients with metabolic co-morbidities (e.g., insulin sensitising agents, statins, angiotensin converting enzyme inhibitors and angiotensin receptor blockers) have therefore also been tried in patients with NASH. A meta-analysis has determined that metformin does not have a beneficial impact on transaminase levels or liver histology, and promising early results are probably attributable to lifestyle intervention in the treatment groups [357,358]. Thiazolidinediones have been trialed showing some improvement in steatosis and inflammation and reduced fibrosis progression [357–360]. A recent study, published in abstract form only, of 18 months of pioglitazone in diabetic or pre-diabetic patients with biopsy-proven NASH demonstrated a significant reduction in fibrosis *versus* placebo in addition to other markers of disease activity [360]. Recommending these drugs, however, is difficult as they have significant side-effects such as weight gain and, in the case of rosiglitazone, increased risk of myocardial infarction [361]. Furthermore discontinuation of these medications has resulted in worsening steatosis and inflammation suggesting they require long-term use [362]. Statin therapies appear to have some beneficial effects on liver histology [363–366]—while other forms of lipid-lowering therapy probably do not [367]—although high quality data as a specific treatment modality in NASH are lacking. It is safe, however, to recommend the use of statins in NAFLD patients with dyslipidaemia as they do confer a benefit in terms of cardiovascular morbidity and mortality and patients with NASH do not appear to be at increased risk of hepatotoxicity [324,368]. Early studies have shown the use of telmisartan, an angiotensin II receptor blocker (ARB) with PPAR-γ-modulating activity, is associated with some improvement in fibrosis in hypertensive patients with NASH [369] and 48 weeks of losartan, another ARB, induced a significant reduction in serum fibrosis markers and numbers of activated HSCs [370,371]. These findings have not been replicated. Currently there is insufficient evidence to support the use of these medications as specific treatments for NAFLD, although the aggressive management of cardiovascular risk factors is recommended [324,372].

## 12. Summary and Conclusions

The pathogenesis of NASH, and most importantly the progression of fibrosis, is a complex process that occurs in response to a chronic inflammatory state in the setting of obesity, insulin resistance, hepatic steatosis and oxidative stress. The appearance of portal fibrosis, typically accompanied by a chronic inflammatory infiltrate, characterises more advanced disease and these patients are at risk of progression to cirrhosis and its associated complications. The reasons underlying the development of portal fibrosis in a disease of parenchymal injury are unclear, but it seems apparent that disease progression is much more complex than a sequence of consecutive “hits” of steatosis and inflammation. Furthermore, patterns of fibrosis vary between adults and children with NAFLD, although the disease is ostensibly the same, suggesting that NAFLD is both clinically and pathogenically a heterogeneous disease with multiple fibrogenic pathways.

Our knowledge of the pathogenesis of NASH has been greatly advanced through the examination of liver specimens from patients with NAFLD and animal models of the disease, as well as *in vitro* studies of cellular responses and interactions. Some key findings appear to underpin the current understanding of disease progression. First, excess FFAs (especially SFAs) and the generation of toxic metabolites due to increased FFA traffic appear to be central to development of oxidative stress that leads to hepatocyte injury—a process known as lipotoxicity. Insulin resistance is also critically important and both predisposes to damage and is exacerbated by the inflammatory milieu of obesity and NAFLD. Cellular injury leads to hepatocyte apoptosis, which represents an important nexus with the propagation inflammation and activation of pro-fibrogenic HSCs, as well as hepatocyte senescence, which may contribute to pathogenesis through the recruitment of immune mediators (particularly macrophages) that guide disease progression. In the absence of an obvious antigen, NASH appears to involve a sterile inflammatory response with the key mediators being TLRs (especially TLR4 and 9) and cytokines (especially TGFβ and IL-1β) that promote inflammation and fibrogenesis. With chronic liver injury an alternative regenerative pathway is activated that involves expansion of a compartment of HPCs and the appearance of an associated DR. These events correlate with severity of injury in chronic liver diseases and the progression of fibrosis, although their roles in fibrogenesis are still being explored. It is likely that the portal inflammatory infiltrate mediates the progression of disease and is critical to the development of fibrosis, possibly through direct interaction with elements of the DR and HPC compartment. Recent studies also suggest that portal fibrogenesis in NASH is associated with, and potentially mediated by, a Th17 response via increased expression of IL-1β, TGFβ, and IL-6. Therapies for NASH to this point have been disappointing but have progressed beyond merely treating hepatic steatosis as a precursor to inflammation. Antioxidants have yielded conflicting results suggesting that an approach that addresses the inflammatory response will become central to any successful treatments for NASH and progressive fibrosis. It is hoped that an increased understanding of the mechanisms mediating disease progression, and particularly the nature of the immune response and key cellular interactions, will provide better targets for therapeutic intervention in this increasingly common disease.

## Figures and Tables

**Table 1.** Features of senescent cells.

Cell Parameter	Features of Senescent Cells
Cell cycling	Permanent, irreversible cell cycle arrest

Mechanisms of senescence	Critical telomere loss inducing a DDR-mediated growth arrest
	Genomic damage (especially DNA double-strand breaks)
	Oncogene-induced senescence
	Stress-induced senescence (e.g., oxidative stress, serum-depletion *in vitro*)

Cell morphology	Irregular shape and increased size

Markers of senescence	Increased beta-galactosidase activity and expression
	Increased p21^WAF1^, p16^INK4A^, p15^INK4B^, p53 and RB expression
	Decreased expression of Ki-67, cyclin A and CDK2
	Formation of SAHF and SDF (e.g., HP1β, γ-H2A.X)
	Formation of DNA-SCARS (reflecting DDR or telomere dysfunction)

Cellular activity	Permanent growth arrest but metabolically active
	Production of factors with autocrine/paracrine activity
	**the senescence-associated secretory phenotype (SASP)**

Abbreviations: DDR, DNA-damage response; DNA, deoxyribonucleic acid; RB, retinoblastoma; CDK2, cyclin-dependent kinase 2; SAHF, senescence-associated heterochromatin foci; SDF, senescence-associated damage foci; HP1β, heterochromatin protein-1-beta; γH2AX, gamma-histone 2AX; DNA-SCARS, DNA segments with chromatin alterations reinforcing senescence; SASP, senescence-associated secretory phenotype.
